# A Systematic Review and Meta-Analysis on the Relationship between Body Dissatisfaction and Morbid Exercise Behaviour

**DOI:** 10.3390/ijerph18020585

**Published:** 2021-01-12

**Authors:** Manuel Alcaraz-Ibáñez, Adrian Paterna, Álvaro Sicilia, Mark D. Griffiths

**Affiliations:** 1 Health Research Centre and Department of Education, University of Almería, 04120 Almería, Spain; m.alcaraz@ual.es (M.A.-I.); asicilia@ual.es (Á.S.); 2 Psychology Department, Nottingham Trent University, Nottingham NG1 4FQ, UK; mark.griffiths@ntu.ac.uk

**Keywords:** problematic exercise, exercise dependence, exercise addiction, body image, body shape, body dissatisfaction

## Abstract

Background: The present study aimed to quantify the relationship between body dissatisfaction and morbid exercise behaviour (MEB). Methods: The electronic databases MEDLINE, PsycINFO, Web of Science, SciELO, and Dissertations & Theses Global were searched from inception to September 2020. Pooled effect sizes corrected for sampling errors (*r^+^*) were computed using a bare-bones meta-analysis. The robustness of the results was examined by influence analyses. The presence of moderators was examined by inspection of the variance in *r^+^* attributable to sampling errors and 80% credibility intervals, followed by subgroup analysis and univariable/multivariable meta-regressions. Publication bias was examined by visual inspection of funnel plot symmetry, cumulative meta-analysis, and Egger’s test. Results: A total of 41 effect sizes from 33 studies (*n* = 8747) were retrieved. Results showed a significant and near to moderate effect size (*r^+^* = 0.267, 95% CI = 0.226 to 0.307), and this did not differ by gender, BMI, age, percentage of Whites, study quality, or MEB measure. Conversely, effect sizes were found to be stronger in published and more recently conducted studies. Conclusion: The findings indicate that body dissatisfaction is one of the likely causes underlying MEB. This suggests the need for further longitudinal research aimed at confirming the potential causal nature of this relationship.

## 1. Introduction

Exercise is defined as a planned, structured, and repetitive sub-form of physical activity aimed at improving fitness and health [1]. However, research has shown that for a minority of individuals, exercise may turn into a non-necessarily healthy and even problematic behaviour [2]. For instance, when exercising interferes with individuals’ social relationships or professional obligations, or when the impossibility of engaging in exercise results in increased depression and/or anxiety symptoms [3,4]. Irrespective of the multiplicity of terms used to refer to this kind of behaviour (e.g., compulsive exercise, exercise dependence, exercise addiction, etc.) [5], the common element underlying the phenomenon under consideration (which in the present study will be generically referred to as morbid exercise behaviour; MEB) [6,7] is that exercise becomes increasingly uncontrollable, therefore becoming a source of physical and/or psychological harm [2]. In view of these considerations, it is necessary to clarify the mechanisms involved in the emergence of this unhealthy form of exercise.

According to psychological models, MEB may be explained by individual differences in the goals and expectations associated with engaging in the behaviour [4], such as those concerning the improvement of body attributes [8,9,10]. This is not surprising given that body dissatisfaction (i.e., evaluating one’s own body negatively) [11] is a very common experience across different populations [5,12], as well as the potential that exercise has to modify body features [13].

Two different mechanisms may be involved in the process whereby experiencing body dissatisfaction may lead to MEB [4]. Firstly, there is a negative reinforcement mechanism, which in this context implies that exercising is fuelled to avoid negative body-related consequences that may emerge as a result of not engaging in the behaviour (e.g., feeling guilty about missing an exercise session and losing an opportunity to compensate for caloric intake), an action that may subsequently translate into increased body fat. Secondly, there is a positive reinforcement mechanism, which implies exercising in the hope of obtaining a body-related pleasurable reward (e.g., increased muscle tone) [2].

To date, numerous studies have examined the association between body dissatisfaction and MEB e.g., [14,15,16,17]. Overall, findings from these studies suggest that body dissatisfaction and MEB are positively associated. However, estimates of the association between these two variables have been found to vary widely across studies [18,19,20], without the reasons for these differences having been examined by employing meta-analytic techniques. Gaining deeper insight into the relationship between body dissatisfaction and MEB, and further considering the factors that may account for such a relationship, could contribute to guide professional practice of exercise and health practitioners. Additionally, identifying possible gaps in the extant literature may inform future research concerning the aetiology of MEB. Therefore, the present study had a two-fold objective. Firstly, to quantify the magnitude of the relationship between body dissatisfaction and MEB. Secondly, to explore potential demographic and methodological moderators of this relationship.

## 2. Method

The present systematic review and meta-analysis was conducted in accordance with the Preferred Reporting Items for Systematic Reviews and Meta Analyses (PRISMA) checklist [21] (see Table S1 in supplementary material).

### 2.1. Locating Studies

The electronic bibliographic databases MEDLINE, PsycINFO, Web of Science, SciELO, and Dissertations & Theses Global were searched for eligible studies from inception to September 2020 using the following search strategy: (“problematic exercise” OR “morbid exercise” OR “exercise addiction” OR “exercise dependence” OR “compulsive exercise” OR “ compulsive physical activity “ OR “obligatory exercise” OR “commitment to exercise” OR “excessive exercise”) AND (dissatisfaction OR “body dissatisfaction” OR “body shape” OR “body image”). No geographical or cultural restrictions were applied. Search was limited to studies written in English or Spanish (the languages spoken by the research team; see Table S2 in supplementary material). Reference lists of studies included in the review were manually inspected to identify any further potentially eligible studies.

The references of the retrieved studies were managed in Endnote X9. Studies were independently selected by the first two authors in two stages by examining: (a) titles and abstracts, and (b) full texts. Disagreements were discussed and resolved on a consensual basis with the assistance of a third author when this was required.

When relevant information for a given retrieved study published within the last five-year period was missing (e.g., BMI or age), this was requested from the corresponding authors of the study. When no response was received within a one-month period, the authors were contacted again on one final occasion. The percentage of authors that provided data (after being asked) was 49%.

### 2.2. Defifinition of Morbid Exercise Behaviour and Measure Criteria

The research team agreed to consider eligible studies employing any of the measures considered in a recent previous meta-analytic research examining the correlates of MEB [6] based on the following definition of this construct: “An increasingly uncontrollable exercise-related behaviour that, regardless of the effective time spent exercising, involves physical and/or psychological harm” [2,6].

### 2.3. Eligibility Criteria

The present study collated data on the association between body dissatisfaction and MEB as assessed by self-report instruments. For the purpose of avoiding publication bias, not only were quantitative data from published studies retrieved but also data from unpublished literature (e.g., doctoral dissertations or non-significant findings excluded from publications).

#### 2.3.1. Inclusion Criteria

Studies were considered eligible if the following criteria were met: (a) at least one validated self-report instrument assessing MEB was used; (b) validated instruments assessing body dissatisfaction were used; (c) were written in English or Spanish (although no restrictions in terms of country of origin were considered); and (d) sufficient data to compute effect size were available.

#### 2.3.2. Exclusion Criteria

Studies were excluded on the basis of the following criteria if the study: (a) had an experimental methodology; (b) assessed body dissatisfaction employing self-reported instruments that (i) did not reflect global experiences of body dissatisfaction (e.g., those focused on specific features or parts of the body); (ii) were based on body silhouettes, because these measures do not necessarily reflect a negative evaluation of the current body [22]; and/or (iii) only assessed evaluations of a positive nature because these do not necessarily imply experiencing low levels of dissatisfaction [23]; (c) only had composite scores comprising two or more instruments assessing MEB which meant individual scores derived from each instrument were not available; (d) only addressed MEB in terms of exercise intensity or volume (e.g., frequency or hours of practice within a given period); (e) had specific items or factors excluded when obtaining global scores for body dissatisfaction or MEB, provided that scores concerning specific factors or components were not available; (f) had scores for the constructs under consideration that comprised: (i) a factorial structure that differed from the one originally proposed, (ii) isolated items extracted from validated questionnaires, or (iii) composite scores derived from more than one psychometric scale; (g) had a study population comprising professional athletes; and (h) had a sample size below 30 individuals.

### 2.4. Coding Procedure

After reviewing the common features of the studies retrieved in a preliminary search, a coding frame was developed and pilot-tested. This coding sheet was used by the first two authors of the present study when extracting data from the retrieved studies (see List S1 in supplementary material). Disagreements between both authors were discussed and resolved on a consensual basis with the assistance of a third author when this was required. The following coding categories were considered: (a) citation and year of publication; (b) sample size; (c) gender; (d) age; (e) BMI; (f) % of Whites; (g) body dissatisfaction measure; (h) MEB measure; (i) publication status; (j) reporting of leisure-time exercise; (k) regular exercisers (i.e., they engaged in exercise at least once a week; [24]); (l) study quality; (m) study design; and (n) effect size of the correlation between body dissatisfaction and MEB. These coded features were used for descriptive purposes and, where appropriate, as potential moderator variables [25].

### 2.5. Risk of Bias

The adapted Newcastle–Ottawa Scale (NOS) for evaluating cross-sectional/survey studies [26] was employed for the assessment of risk bias. The NOS is scored on a 0–16 range based on the following components: (a) clarity of stated aim; (b) representativeness of the sample; (c) sample size; (d) non-respondents; (e) ascertainment of the exposure; (f) control of confounding factors; (g) comparability of participants in different outcome groups; (h) assessment of the outcome; and (i) statistical tests. The assessment of risk bias was independently conducted by the first two authors of the present study. Disagreements between both authors were discussed and resolved on a consensual basis with the assistance of a third author if necessary. As a result of this procedure, the 33 retrieved studies were scored between 7 and 11 in terms of risk of bias.

### 2.6. Statistical Analysis

#### 2.6.1. Effect Size Calculations

Pearson’s correlation (*r*) was employed as the effect size index. In the case of studies providing effect sizes considering only subdomains of a given instrument, e.g., [14], these were joined to allow for obtaining also effect sizes corresponding to global scores. In the case of studies that provided effect sizes using Cohen’s *d*, e.g., [16], these were transformed into an *r*-score.

Since information concerning reliability was missing in many of the studies included in the present meta-analysis, the relationship between body dissatisfaction and MEB was quantified using a methodological approach (i.e., bare-bones meta-analysis) that allows for obtaining pooled effect sizes corrected for sampling errors (*r^+^*) [27]. The random-effects model used in this procedure assumes that variations in the distribution and sampling errors of effect sizes may contribute to explain differences between them [28]. A given *r^+^* value was judged to be statistically significant when its 95% confidence interval (CI) did not contain zero.

Statistical heterogeneity was assessed using *I^2^* statistic, assuming that values of 25%, 50%, and 75% suggest low, moderate, and high heterogeneity, respectively [29]. The robustness of the results was examined by inspecting potential outliers using influence analyses (DIFITS, Cook’s distance, and COVRATIO statistics) [30]. Findings from these analyses also served to examine whether a particular study may be accounting for a large proportion of heterogeneity.

The presence of significant moderators was examined by inspection of (i) the variance in *r^+^* attributable to sampling errors in the pooled effect size (i.e., statistical artefacts, *Var_art_%*) and (ii) 80% credibility intervals (i.e., 80% CV). Values of *Var_art_%* below 75% or the presence of wide 80% CV were considered as indicative of the existence of significant moderators [27]. Provided that at least four effect sizes were available [31], analogue to ANOVA analyses were employed to examine statistical significance of between-group difference in effect size moderator analyses [32] for the following categorical variables: gender, publication status, MEB measure, and reporting of leisure-time exercise levels. Provided that at least ten effects sizes were available, both continuous covariates (i.e., BMI, age, % of Whites, study quality, and publication year) and categorical variables (transformed into dummy variables) were examined as potential sources of variance in heterogeneity using a mixed-effects model meta-regressions [31]. Meta-regressions were firstly conducted by employing univariable models (i.e., considering each potential moderator in isolation) and then, by employing multivariable models in which all significant moderators identified in the first stage were simultaneously introduced. Explained variance by the moderators was quantified as a percentage and expressed by *R^2^*. Provided that at least ten effect sizes were available [33], publication bias was examined by visual inspection of funnel plot symmetry, cumulative meta-analysis, and Egger’s test (*p* > 0.10).

Point mean estimates of effect sizes were interpreted as follows: values from 0.00 to 0.10 suggest trivial effect; from 0.10 to 0.30 small effect; from 0.30 to 0.50 moderate effect; and >0.50 large effect [34]. The described statistical analyses were estimated using Hunter–Schmidt method (i.e., dividing by k − 1 rather than k) in R environment with the “Psychmeta” package [35].

#### 2.6.2. Dependence

The fact of considering multiples effect sizes from a single sample may have led to generating dependence [36,37]. To avoid this, the following actions were taken when (a) MEB was assessed using multiple instruments [38], and given that subgroup analyses according to this feature were planned, random removal of effect sizes was conducted until just one effect size remained [39]; (b) different effect sizes were provided for several groups in a same study (e.g., men/women), each of these was treated individually [38]; and (c) the relationship between body dissatisfaction and MEB was examined over time (i.e., in longitudinal studies), the dependent effect sizes were averaged within each study before conducting the analysis [39].

## 3. Results

### 3.1. Selection and Description of Studies

**Figure 1:**
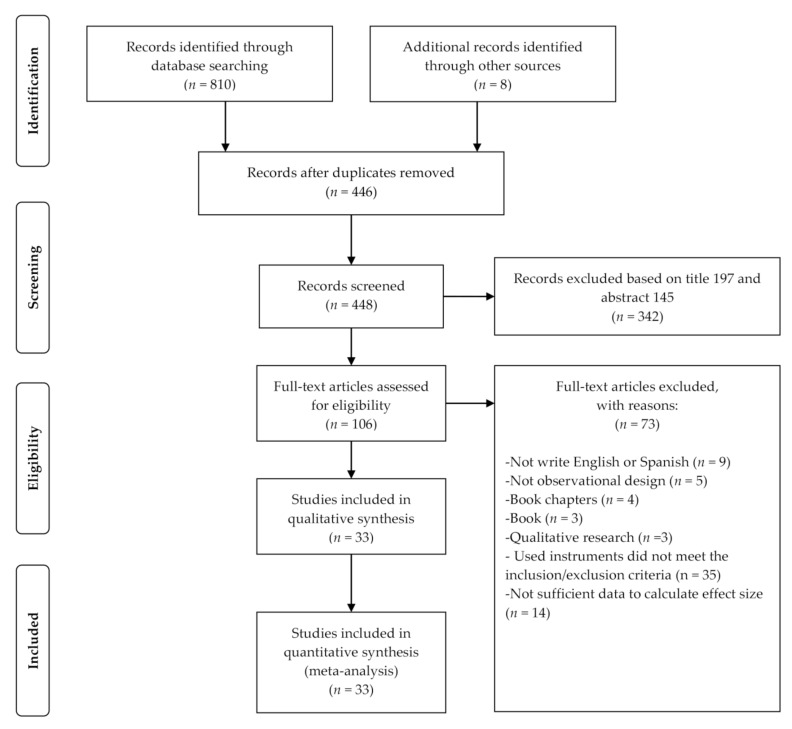
PRIMA flow diagram of study selection.

A total of 810 studies were identified from multiple database search. As a result of the study selection procedure (see Figure 1), 33 studies comprising 41 effect sizes (*n* = 8747) were included in the systematic review and meta-analysis (see Table 1). From the studies included in the meta-analyses, 26 were published in peer-reviewed papers and seven were published in doctoral theses. These were conducted between 1995 and 2020. The instruments employed for the assessment of MEB were the Commitment Exercise Scale (CES; *K* = 12), Compulsive Exercise Test (CET; *K* = 6), Exercise Dependence Scale-Revised (EDS-R; *K* = 10), and Obligatory Exercise Questionnaire (OEQ; *K* = 13). The instruments employed for the assessment of MEB were the Body Shape Questionnaire (BSQ; *K* = 14), Eating Disorders Inventory (EDI; *K* = 4), Eating Disorders Inventory-2 (EDI-2; *K* = 16), Eating Disorders Inventory-3 (EDI-3; *K* = 1), Body Dissatisfaction Subscale of the Eating Pathology Symptoms Inventory (EPSI; *K* = 1), Male Body Attitudes Scale (MBAS; *K* = 4), and Body Areas Satisfaction Subscale of the Multidimensional Body-Self Relations Questionnaire (MBSRQ-AS; *K* = 1). A total of 18 studies reported that their samples comprised regular exercisers whereas 23 studies did not report information on this matter. The mean age and BMI of the participants included in the samples retrieved in the present meta-analyses, respectively, ranged from 13.02 to 36.00 years (*M_age_* = 23.66, *SD_age_* = 7.19) and from 19.30 to 25.49 kg/m^2^ (*M_BMI_* = 22.58, *SD_BMI_* = 1.42).

### 3.2. Body Dissatisfaction and MEB

**Figure 2:**
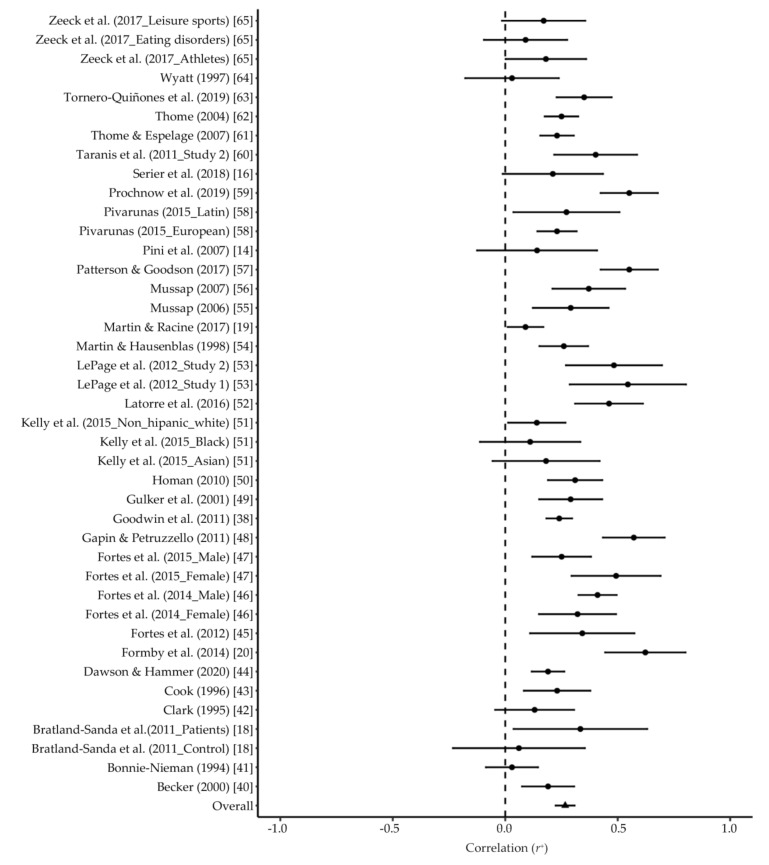
Forest plots of relationship between MEB and body dissatisfaction.

The analysis examining the relationship between body dissatisfaction and MEB (see Figure 2) included 41 effect sizes from 33 studies (*N_total_* = 8747). Findings from the bare-bones meta-analysis showed a significant near to moderate effect size (*r^+^* = 0.267, 95% CI = 0.226 to 0.307). The high heterogeneity (*I^2^* = 77.1), along with the values of *Var_art_%* (5.8) and 80% CV (0.117 to 0.417), suggested the presence of potential moderators. Findings from analog to ANOVA analyses (see Table 2) showed significant differences across groups according to (a) publication status [*F*(1, 19) = 6.510], the effect size being lower for unpublished (*K* = 8; *r^+^* = 0.193, 95% CI = 0.138 to 0.248) than published (*K* = 33; *r^+^* = 0.288, 95% CI = 0.240 to 0.335) studies; and (b) body dissatisfaction measure [*F*(3, 10) = 11.6], with effect-sizes ranging from low for MBAS (*K* = 4; *r^+^* = 0.173, 95% CI = 0.147 to 0.199), EDI (*K* = 4; *r^+^* = 0.223, 95% CI = 0.001 to 0.445) and EDI-2 (*K* = 16; *r^+^* = 0.238, 95% CI = 0.210 to 0.267) to moderate for BSQ (*K* = 14; *r^+^* = 0.379, 95% CI = 0.318 to 0.441). Conversely, no significant differences between groups were found according to gender [*F*(2, 17) = 0.254], reporting of leisure-time exercise [*F*(1, 38) = 1.94], regular exercisers [*F*(1, 24) = 3.28] and MEB measure [*F*(3, 16) = 0.503]. After removing effect sizes for which no mean BMI (*K* = 14), age (*K* = 3), or % of Whites (*K* = 20) were available, findings from the univariable meta-regression analysis (see Table 3) showed publication status, body dissatisfaction measure and year of publication to be significant continuous moderators of the relationship between body dissatisfaction and MEB. More specifically, the relationship under consideration was stronger in published versus unpublished studies, as well as in more recently published studies. Furthermore, the findings from the multivariable meta-regression analysis (see Table 3) showed that the three significant moderators emerged in the univariable meta-regression analysis together (i.e., publication status, body dissatisfaction measure and year of publication) explained 64.18% of the variance of the relationship between body dissatisfaction and MEB.

### 3.3. Sensitivity Analysis and Publication Bias

The results of influence analyses supported the robustness of the findings concerning the relationship between body dissatisfaction and MEB (see Figure S1 in supplementary material). Funnel plot symmetry (see Figure S2 in supplementary material) and the results of cumulative meta-analysis showed that the inclusion of studies with high standards errors and small sample sizes did not significantly affect the pooled effect sizes (see Figure S3 in supplementary material In addition, the results of the Egger test did not suggest the presence of publication bias (*p* = 0.773).

## 4. Discussion

The present study is the first to provide evidence on the relationship between body dissatisfaction and MEB using meta-analytic techniques. Results derived from 41 effect sizes from 33 studies consisting of 8747 participants showed a small but near to moderate positive relationship between body dissatisfaction and MEB. Additionally, the relationship under consideration was not found to differ across male and female individuals, nor to depend on variables such as BMI, age, % of Whites in the sample, study quality, or MEB measure. Conversely, the results showed that the relationship between body dissatisfaction and MEB tends to be stronger in published and more recently conducted studies. These findings extend current knowledge on MEB by quantifying the relationship between this unhealthy form of exercise and one of its potential antecedents. The main implications drawn from the results obtained are discussed below.

### 4.1. Overall Effects

Consistent with psychological models of MEB [4], the results here reinforce the notion that the body improvement-related goals and expectations underlying body dissatisfaction experiences may play an important contributory role on the emergence of MEB [8,9,10]. Findings from the present study also extend the number of possible pathology-related outcomes of body dissatisfaction for which there is evidence at the meta-analytical level. In particular, by suggesting that body dissatisfaction is a maladaptive cognitive procedure not just in terms of leading to the emergence of eating disorders [66] and mood disorders [67] but, additionally, for its likely contribution to the onset and maintenance of another potentially dysfunctional outcome such as MEB.

However, it should be noted that the pathological nature of MEB may not been in not on a par with that of mood disorders and eating disorders. In particular, because it has been suggested that healthy exercise and MEB (at least as operationalised in the currently available assessment instruments) may share some of their attributes [68,69]. Indeed, this circumstance has led some authors to suggest the need to control by exercise volume or even by perceived health status when examining the relationship between MEB and its potential antecedents [69,70,71]. However, it should be noted that these two factors were not taken into account when computing the original effect sizes retrieved in the present meta-analysis. Consequently, the possibility exists that the magnitude of the relationship between body dissatisfaction and truly MEB would be effectively weaker.

The magnitude of the relationship between body dissatisfaction and MEB found in the present study appears to be slightly lower than the one reported in the only meta-analysis to date that has investigated the relationship between other body-related variables and MEB [72]. This circumstance could be due to differences between the body-related construct (i.e., drive for muscularity) considered in the study whose results are being used for comparison [72] and body dissatisfaction. Firstly, experiencing a drive for muscularity does not necessarily imply individuals as being globally dissatisfied with their bodies but is the desire of having a more muscular physique [73]. Similarly, it has been suggested that muscular development is perceived as a more easily attainable feature than others that, such as thinness, may be involved in global experiences of the body [74].

Following the assumption that positive reinforcement mechanisms are present in the development of MEB [4], the possibility therefore exists that the differences favouring drive for muscularity versus body dissatisfaction may be due to the easily attainable nature of the gains inherent to the former experience. These differences may also be explained by the fact that drive for muscularity construct involves a behavioural component (i.e., acting upon the desire of having a more muscular physique) not present in body dissatisfaction [73], in particular, if considering the primarily behavioural nature of MEB [2,4]. The aforementioned explanation may be even more feasible if it is considered that, unlike the present study, the population in the study examining the relationship between drive for muscularity and MEB used here for comparison purposes comprised male individuals [72], who have been reported to pursue muscularity and engagement concerning strategies aimed at achieving this goal to a greater extent than their female counterparts [75]. In view of these considerations, further research is needed that provide a better understanding of the role that the very precise nature of the different body-related experiences may have concerning the aetiology of MEB.

### 4.2. Moderators of the Relationship between Body Dissatisfaction and MEB

The fact that neither gender, BMI, age, nor ethnicity (expressed as % of Whites) emerged as significant moderators of the relationship between body dissatisfaction and MEB, suggest that such a relationship may be largely consistent across individuals with different sociodemographic characteristics. The fact that no significant differences in the relationship under consideration were found according to the instrument employed for the assessment of MEB suggests that experiencing body dissatisfaction is consistently associated with the different sets of components involved in each of these measures irrespective of the ones adapted from the clinical criteria for substance dependence [76] or those proposed as maintenance factors for excessive exercise within the ED domain [77]. These two groups of findings are noteworthy if it is assumed that body dissatisfaction may preclude MEB in time. In particular, because this would imply that focusing on managing body dissatisfaction may be a largely universal effective strategy to prevent the emergence of MEB in its different forms. For its part, the fact that the magnitude of the relationship between body dissatisfaction and MEB increased linearly with time suggests that exercise may be increasingly employed as a form of coping with body modification. However, this is proposed as just one possible interpretation of the finding here, and whose empirical validity should be tested in further studies. Finally, a plausible explanation for the weaker relationship found between body dissatisfaction and MEB when the former is assessed with the MBAS may be due to the distinctive characteristics of this instrument. In particular, it should be noted that one of the characteristics present in the MBAS (i.e., height) does not appear to be susceptible to improvement through physical exercise. In absence of this potential for improving, it appears plausible to assume that the positive reinforcement obtained from exercising could be tempered.

### 4.3. Practical Implications

The results of the present study suggest that exercise professionals such as personal trainers or fitness instructors should be aware of the risk that experiencing high levels of body dissatisfaction would represent in terms of developing MEB. Consequently, exercise professionals (e.g., exercise managers and fitness instructors) and primary healthcare providers may be encouraged to respectively move away from using exercise primarily as a body shape change or weight loss tool [13,78]. The results presented here also open the door to explore the possibility that implementing intervention programmes of a psycho-educational nature aimed at decreasing body dissatisfaction [79] among recreational exercisers may contribute to the prevention of the occurrence of MEB and, by extension, to enhance the potential health-inducing character of exercise.

### 4.4. Limitations

Findings from the present meta-analysis should be interpreted in the light of several limitations. Firstly, it is likely that variables not examined in the present study due to the unavailability of data could operate as moderators of the relationship under consideration. A clear example would be the clinical nature of the sample in terms of eating disorders. In particular, since associations between MEB and variables involving a negative evaluation of the body have been found to be weaker among individuals at high-risk relative to those at low-risk of developing eating disorders [70]. Therefore, further research aimed at examining the relationship between body dissatisfaction and MEB among individuals at high-risk of eating disorders appears warranted.

Secondly, the lack of reporting on scores for the different factors included in the instruments assessing MEB prevented the authors from examining the extent to which these may be differentially associated with body dissatisfaction. This limitation is of particular relevance in the light of evidence suggesting that (i) instruments assessing MEB may be better operationalised from a multi-dimensional perspective [20,80], and (ii) the strength of the associations between MEB and its potential antecedents may largely vary across the specific components of the former [6,81]. In view of these implications, researchers in this field are encouraged to examine the associations and links between body dissatisfaction and MEB considering the latter not just as a global phenomenon but also according to its specific components. Moreover, there is a need to examine the relationship between body dissatisfaction and MEB that considers the individual components of MEB. This is necessary because some of the MEB components may reflect exercise motives related to body dissatisfaction (e.g., exercising to control weight) [6].

Finally, the fact that the retrieved data were largely cross-sectional does not allow the drawing of firm conclusions regarding causality based solely in the present study’s findings. In view of this limitation, and given the theoretical plausibility of considering body dissatisfaction as a potential antecedent of MEB [2,4], research featuring longitudinal designs are needed that provide further insight into the hypothetical causal nature of the relationship between body dissatisfaction and MEB. A feasible possibility in this respect would be examining the extent to which repeated acute exercise-induced changes in state-level of body dissatisfaction assessed may lead to long-term changes in the levels of MEB.

## 5. Conclusions

In brief, findings from the present meta-analysis contribute to the understanding of the aetiology of MEB by pointing to body dissatisfaction as one of the likely causes underlying this form of non-healthy exercise behaviour. The fact that the small but near to moderate relationship found between body dissatisfaction and MEB tends to be more pronounced in recently published papers raises the need for further research on this topic. In view of the gaps identified in the present study, these research efforts may benefit from (i) employing longitudinal designs; (ii) examining the variables that may be accounting for this relationship; (iii) considering the different specific components involved in MEB; and (iv) considering different population groups (including clinical populations in terms of risk of eating disorders). Such research may provide better understanding on the very specific circumstances under which experiencing body dissatisfaction may lead to MEB and, therefore, to compromise the potential health-related benefits of exercise.

## Figures and Tables

**Table 1:** Characteristics and effect size of the relationship between body dissatisfaction and morbid exercise behaviour.

Study	*n*	Gender	Age	BMI	Reporting of Leisure-Time Exercise	Regular Exercisers	% of Whites	BD Measure	MEB Measure	Publication Status	Study Quality	Study Design	ES (*r*)
Becker (2000) [40]	250	Female	20.70	-	Yes	Unknown	76.00	EDI-2	OEQ	Unpublished	8	Cross-sectional	0.19
Bonnie-Nieman (1994) [41]	250	Mixed	14.61	22.04	Yes	Unknown	68.00	EDI	OEQ	Unpublished	9	Cross-sectional	0.03
Bratland-Sanda et al. (2011_Control) [18]	42	Female	31.30	25.30	Yes	Unknown	-	EDI-2	EDS-R	Published	11	Cross-sectional	0.06
Bratland-Sanda et al. (2011_Patients) [18]	41	Female	30.10	20.90	Yes	Unknown	-	EDI-2	EDS-R	Published	11	Cross-sectional	0.33
Clark (1995) [42]	111	Female	19.04	22.82	Yes	Yes	92.00	EDI-2	OEQ	Unpublished	9	Cross-sectional	0.13
Cook (1996) [43]	155	Female	34.90	-	Yes	Yes		EDI	CES	Unpublished	8	Cross-sectional	0.23
Dawson and Hammer (2020) [44]	632	Male	28.31	-	No	Unknown	78.50	MBAS	EDS-R	Published	10	Cross-sectional	0.19
Formby et al. (2014) [20]	107	Mixed	14.90	-	No	Unknown	-	EDI-3	CET	Published	9	Cross-sectional	0.62
Fortes et al. (2012) [45]	65	Mixed	16.10	-	Yes	Unknown	-	BSQ	CES	Published	9	Cross-sectional	0.34
Fortes et al. (2014_Female) [46]	116	Female	14.54	20.43	Yes	Yes	80.20	BSQ	CES	Published	11	Cross-sectional	0.32
Fortes et al. (2014_Male) [46]	464	Male	15.02	21.29	Yes	Yes	62.20	BSQ	CES	Published	11	Cross-sectional	0.41
Fortes et al. (2015_Female) [47]	88	Female	-	-	Yes	Yes	68.18	BSQ	CES	Published	10	Cross-sectional	0.49
Fortes et al. (2015_Male) [47]	198	Male	-	-	Yes	Yes	50.56	BSQ	CES	Published	10	Cross-sectional	0.25
Gapin and Petruzzello (2011) [48]	179	Mixed	35.88	22.71	Yes	Yes	-	EDI	OEQ	Published	9	Cross-sectional	0.57
Goodwin et al. (2011) [38]	1012	Mixed	13.02	-	Yes	Unknown	94.70	EDI-2	CET	Published	9	Cross-sectional	0.24
Gulker et al. (2001) [49]	172	Mixed	36.00	-	Yes	Yes	-	EDI-2	OEQ	Published	9	Cross-sectional	0.29
Homan (2010) [50]	231	Female	19.20	22.00	No	Unknown	97.00	MBSRQ-AS	OEQ	Published	9	Longitudinal	0.31
Kelly et al. (2015_Asian) [51]	62	Male	19.32	22.34	No	Unknown	0.00	MBAS	EDS-R	Published	10	Cross-sectional	0.18
Kelly et al. (2015_Black) [51]	70	Male	19.71	25.49	No	Unknown	0.00	MBAS	EDS-R	Published	10	Cross-sectional	0.11
Kelly et al. (2015_Non_hipanic_white) [51]	208	Male	19.63	23.58	No	Unknown	100.00	MBAS	EDS-R	Published	10	Cross-sectional	0.14
Latorre et al. (2016) [52]	149	Mixed	32.10	23.72	Yes	Yes	-	BSQ	EDS-R	Published	11	Cross-sectional	0.46
LePage et al. (2012_Study 1) [53]	53	Female	19.06	-	Yes	Yes	90.20	BSQ	OEQ	Published	9	EMA	0.54
LePage et al. (2012_Study 2) [53]	76	Female	19.08	22.46	Yes	Yes	86.80	BSQ	OEQ	Published	10	Cross-sectional	0.48
Martin and Hausenblas (1998) [54]	286	Female	34.11	-	Yes	Yes	-	EDI-2	CES	Published	9	Cross-sectional	0.26
Martin and Racine (2017) [19]	531	Mixed	19.37	24.00	No	Unknown	90.60	EPSI	CET	Published	10	Cross-sectional	0.09
Mussap (2006) [55]	120	Male	25.94	24.32	No	Unknown	-	EDI-2	OEQ	Published	7	Cross-sectional	0.29
Mussap (2007) [56]	130	Female	25.10	-	No	Unknown	-	EDI-2	OEQ	Published	7	Cross-sectional	0.37
Patterson and Goodson (2017) [57]	208	Mixed	-	-	Yes	Unknown	87.00	BSQ	CET	Published	10	Cross-sectional	0.55
Pini et al. (2007) [14]	50	Mixed	35.40	-	Yes	Yes	-	EDI-2	CES	Published	8	Cross-sectional	0.14
Pivarunas (2015_European) [58]	439	Female	18.67	22.21	No	Unknown	-	BSQ	EDS-R	Unpublished	10	Cross-sectional	0.23
Pivarunas (2015_Latin) [58]	63	Female	18.75	24.68	No	Unknown	-	BSQ	EDS-R	Unpublished	10	Cross-sectional	0.27
Prochnow et al. (2019) [59]	208	Female	19.40	22.10	Yes	Unknown	87.00	BSQ	CET	Published	12	Cross-sectional	0.55
Serier et al. (2018) [16]	70	Female	34.08	23.29	No	Unknown	58.60	BSQ	OEQ	Published	10	Cross-sectional	0.21
Taranis et al. (2011_Study 2) [60]	101	Female	20.90	21.80	Yes	Yes	-	EDI-2	CET	Published	9	Cross-sectional	0.40
Thome and Espelage (2007) [61]	599	Female	20.12	22.00	Yes	Unknown	77.30	EDI-2	OEQ	Published	10	Cross-sectional	0.23
Thome (2004) [62]	599	Female	20.12	22.00	Yes	Unknown	-	EDI-2	CES	Unpublished	8	Cross-sectional	0.25
Tornero-Quiñones et al. (2019) [63]	225	Mixed	34.20	22.12	Yes	Yes	-	BSQ	EDS-R	Published	11	Cross-sectional	0.35
Wyatt (1997) [64]	80	Mixed	30.85	23.23	Yes	Yes	81.30	EDI	OEQ	Unpublished	11	Cross-sectional	0.03
Zeeck et al. (2017_Athletes) [65]	107	Mixed	20.20	21.70	Yes	Yes	-	EDI-2	CES	Published	11	Cross-sectional	0.18
Zeeck et al. (2017_Eating disorders) [65]	100	Mixed	26.10	19.30	Yes	Yes	-	EDI-2	CES	Published	11	Cross-sectional	0.09
Zeeck et al. (2017_Leisure sports) [65]	100	Mixed	23.30	21.80	Yes	Yes	-	EDI-2	CES	Published	11	Cross-sectional	0.17

*Note:* ES (*r*) = Uncorrected correlation; BMI = Body mass index; BD = Body dissatisfaction; MEB = Morbid exercise behaviour; EDI = Body Dissatisfaction Subscale of the Eating Disorders Inventory; EDI-2 = Body Dissatisfaction Subscale of the Eating Disorders Inventory-2; EDI-3 = Body Dissatisfaction Subscale of the Eating Disorders Inventory-3; MBAS = Male Body Attitudes Scale; EPSI = Body Dissatisfaction Subscale of the Eating Pathology Symptoms Inventory; MBSRQ-AS = Body Areas Satisfaction Subscale of the Multidimensional Body-Self Relations Questionnaire; BSQ = Body Shape Questionnaire; CET = Compulsive Exercise Test; CES = Commitment Exercise Scale; EDS-R = Exercise Dependence Scale-Revised; OEQ = Obligatory Exercise Questionnaire.

**Table 2:** Results of subgroups analyses.

Subgroups	*K*	*n*	*r^+^*	*SDr^+^*	95% CI		80% CV		*Var_art_*%	*I* ^2^
					Lo	Up	Lo	Up		
*Gender*										
Male	7	1754	0.253	0.103	0.177	0.329	0.145	0.360	1.17	66.68
Female	19	3654	0.282	0.104	0.235	0.329	0.179	0.384	4.71	59.04
Both	15	3343	0.257	0.170	0.171	0.343	0.055	0.459	1.30	86.23
*Regular exercisers*										
Yes	18	2631	0.306	0.123	0.249	0.362	0.181	0.430	2.18	62.38
Unknown	23	6116	0.250	0.134	0.195	0.305	0.095	0.405	4.94	81.48
*Reporting of leisure-time exercise*										
Yes	29	6084	0.289	0.135	0.240	0.338	0.137	0.442	2.22	77.75
No	12	2663	0.214	0.114	0.150	0.279	0.093	0.335	4.47	68.32
*Publication Status*										
Unpublished	8	1947	0.193	0.080	0.138	0.248	00.129	0.257	3.38	39.67
Published	33	6800	0.288	0.138	0.240	.335	0.131	0.444	4.29	78.39
*Body dissatisfaction measure*										
BSQ	14	2422	0.379	0.118	0.318	0.441	0.254	0.505	2.33	69.15
EDI	4	664	0.223	0.226	0.001	0.445	−0.051	0.497	0.29	89.31
EDI-2	16	3820	0.238	0.059	0.210	0.267	0.238	0.238	10.85	0.00
EDI-3	1	107	0.623	-	0.506	0.739	0.623	0.623	0.39	-
EPSI	1	531	0.090	-	0.006	0.175	0.090	0.090	0.85	-
MBAS	4	972	0.173	0.027	0.147	0.199	0.173	0.173	16.72	0.00
MBSRQ-AS	1	231	0.312	-	0.194	0.427	0.311	0.311	0.57	-
*Morbid exercise behaviour measure*										
CET	6	2167	0.290	0.174	0.151	0.428	0.076	0.503	0.28	92.24
CES	12	2328	0.282	0.093	0.229	0.334	0.197	0.366	3.66	49.49
EDS-R	10	1931	0.233	0.093	0.175	0.291	0.152	0.315	3.52	46.25
OEQ	13	2321	0.258	0.146	0.178	0.337	0.094	0.421	1.90	76.75

*Note*: *r^+^* = Corrected correlation; *SDr*^+^ = Standard Deviation of *r^+^*; Lo = Lower; Up = Upper; CET = Compulsive Exercise Test; CES = Commitment Exercise Scale; EDS-R = Exercise Dependence Scale-Revised; OEQ = Obligatory Exercise Questionnaire; EDI = Body dissatisfaction subscale of the Eating Disorders Inventory; EDI-2 = Body dissatisfaction subscale of the Eating Disorders Inventory-2; EDI-3 = Body dissatisfaction subscale of the Eating Disorders Inventory-3; MBAS = Male Body Attitudes Scale; EPSI = Body dissatisfaction subscale of the Eating Pathology Symptoms Inventory; MBSRQ-AS = Body Areas Satisfaction Subscale of the Multidimensional Body-Self Relations Questionnaire; BSQ = Body Shape Questionnaire.

**Table 3:** Results of meta-regressions analyses.

Moderators		Univariable Analysis						Multivariable Analysis					
	*K*	*β*_0_ (95%CI)	*β_1_* (95%CI)	QE	QM	*p*	*R* ^2^	*β*_0_ (95%CI)	*β_1_* (95%CI)	QE	QM	*p*	*R* ^2^
*Multivariable level*	41							**0.290 (0.165; 0.415)**					
										70.757	44.708	<0.001	64.18
*Gender*	41			177.407	1.300	0.569	0.00						
Male (RC)	7	**0.233 (0.119; 0.347)**	*-*										
Female	19	**0.306 (0.234; 0.378)**	0.073 (−0.062; 0.208)										
Both	15	**0.281 (0.202; 0.360)**	0.048 (−0.091; 0.187)										
*Regular exercisers*	41			172.403	0.092	0.762	0.00						
Yes (RC)	18	**0.292 (0.219; 0.366)**	-										
Unknown	23	**0.277 (0.214; 0.340)**	−0.015 (−0.304; 0.762)										
*Reporting of leisure-time exercise*	41			172.403	0.092	0.762	0.00						
Yes (RC)	29	**0.299 (0.242; 0.355)**											
No	12	**0.248 (0.161; 0.335)**	−0.050 (−0.154; 0.053)										
*Publication Status*	41			163.584	6.130	0.013	15.38						
Unpublished (RC)	8	**0.173 (0.076; 0.271)**	-										
Published	33	**0.312 (0.262; 0.362)**	**0.139 (0.029; 0.249)**						**0.132 (−0.026; 0.237)**				
*Body dissatisfaction measure*	41			93.696	27.141	<0.001	45.73						
BSQ	14	**0.390 (0.323; 0.456)**											
EDI	4	**0.225 (0.104; 0.346)**	**−0.165 (−0.303; −0.027)**						−0.042 (−0.209; 0.125)				
EDI-2	16	**0.233 (0.172; 0.295)**	**−0.056 (−0.247; −0.066)**						**−0.115 (−0.229; −0.002)**				
EDI-3	1	**0.623 (0.364; 0.882)**	0.233 (−0.034; 0.501)						0.219 (−0.025; 0.462)				
EPSI	1	**0.090 (−0.116; 0.296)**	**−0.299 (−0.516; −0.083)**						**−0.320 (−0.504; −0.136)**				
MBAS	4	**0.160 (0.037; 0.283)**	**−0.230 (−0.369; −0.090)**						**−0.251 (−0.375; −0.128)**				
MBSRQ-AS	1	**0.311 (0.086; 0.535)**	−0.079 (−0.313; −0.155)						−0.082 (−0.294; 0.130)				
*Morbid Exercise Behaviour Measure*	41			174.472	2.396	0.494	0.00						
CET (RC)	6	**0.356 (0.244; 0.467)**	-										
CES	12	**0.280 (0.188; 0.373)**	−0.075 (−0.221; 0.070)										
EDS-R	10	**0.239 (0.138; 0.339)**	−0.117 (−0.267; 0.033)										
OEQ	13	**0.277 (0.191; 0.362)**	−0.079 (−0.220; 0.062)										
*Continuous moderators*													
BMI	27	0.712 (−0.313.; 1.738)	−0.020 (−0.066; 0.025)	114.531	0.750	0.386	0.00						
Age	38	**0.270 (0.105; 0.436)**	0.000 (−0.007; 0.007)	152.344	0.000	0.986	0.00						
% of Whites	21	0.173 (−0.055; 0.400)	0.001 (−0.002; 0.004)	119.750	0.744	0.388	0.00						
Quality	41	0.252 (−0.152; 0.656)	0.003 (−0.038; 0.045)	175.594	0.024	0.877	0.00						
Year of publication	41	**0.358 (0.284; 0.433)**	**−0.008 (−0.014; −0.002)**	165.270	6.048	0.014	14.47		−0.003 (−0.011; 0.006)				

*Note*: *β*_0_ = Intercept/mean effect size; *β*_1_ = Estimated regression coefficient; *R^2^ =* Explained variance; RC = Reference category; QE = Value resulting from the test of residual heterogeneity; QM = Value resulting from the test of moderators; BMI = Body Mass Index; CET = Compulsive Exercise Test; CES = Commitment Exercise Scale; EDS-R = Exercise Dependence Scale-Revised; OEQ = Obligatory Exercise Questionnaire; EDI = Body dissatisfaction subscale of the Eating Disorders Inventory; EDI-2 =Body dissatisfaction subscale of the Eating Disorders Inventory-2; EDI-3 = Body dissatisfaction subscale of the Eating Disorders Inventory-3; MBAS = Male Body Attitudes Scale; EPSI = Body dissatisfaction subscale of the Eating Pathology Symptoms Inventory; MBSRQ-AS = Body Areas Satisfaction Subscale of the Multidimensional Body-Self Relations Questionnaire; BSQ = Body Shape Questionnaire; Statistically-significant effects (*p* < 0.05) appear highlighted in bold.

## Data Availability

Data of the retrieved studies are shown in Table 1.

## Supplementary Materials

The following are available online at https://www.mdpi.com/1660-4601/18/2/585/s1, Table S1: PRISMA 2009 Checklist, Table S2: Search Strategy, List S1: Meta-Analysis Coding Sheet, Figure S1: Influence analyses, Figure S2: Funnel plot, Figure S3: Cumulative Meta-analysis.
